# Detection of Abrin-Like and Prepropulchellin-Like Toxin Genes and Transcripts Using Whole Genome Sequencing and Full-Length Transcript Sequencing of *Abrus precatorius*

**DOI:** 10.3390/toxins11120691

**Published:** 2019-11-25

**Authors:** Blake T. Hovde, Hajnalka E. Daligault, Erik R. Hanschen, Yuliya A. Kunde, Matthew B. Johnson, Shawn R. Starkenburg, Shannon L. Johnson

**Affiliations:** 1 Los Alamos National Laboratory, Bioscience Division, Los Alamos, NM 87545, USA; hovdebt@lanl.gov (B.T.H.); hajkis@lanl.gov (H.E.D.); hanschen@lanl.gov (E.R.H.); y.a.kunde@lanl.gov (Y.A.K.); shawns@lanl.gov (S.R.S.); 2 School of Plant Sciences, College of Agriculture and Life Sciences, University of Arizona, Tucson, AZ 85721, USA; mjohnson@ag.arizona.edu

**Keywords:** abrin, pulchellin, full genome sequencing, genome assembly, isoform, Iso-Seq, *Abrus precatorius*, toxin evolution

## Abstract

The sequenced genome and the leaf transcriptome of a near relative of *Abrus pulchellus* and *Abrus precatorius* was analyzed to characterize the genetic basis of toxin gene expression. From the high-quality genome assembly, a total of 26 potential coding regions were identified that contain genes with abrin-like, pulchellin-like, and agglutinin-like homology, with full-length transcripts detected in leaf tissue for 9 of the 26 coding regions. All of the toxin-like genes were identified within only five isolated regions of the genome, with each region containing 1 to 16 gene variants within each genomic region (<1 Mbp). The *Abrus* *precatorius* cultivar sequenced here contains genes which encode for proteins that are homologous to certain abrin and prepropulchellin genes previously identified, and we observed substantial diversity of genes and predicted gene products in *Abrus precatorius* and previously characterized toxins. This suggests diverse toxin repertoires within *Abrus*, potentially the results of rapid toxin evolution.

## 1. Introduction

Abrin is a toxin classified as a select agent by the United States (US) Department of Health and Human Services [1], and this toxin and its near relatives have been characterized across multiple studies [2,3,4,5,6,7]. Abrin, which acts as a ribosome inhibitory protein, is produced by the rosary pea plant *Abrus precatorius*. A similar toxin of the same family, pulchellin, is produced by a different member of this genus, *Abrus pulchellus*. Both toxins are recognized as type 2 ribosome-inactivating proteins (RIPs)—type II RIPs consist of a single A and single B chain that are linked by a disulfide bond [5]. The A-chain contains the ribosome-inactivating domains, while the B-chain consists of a carbohydrate binding domain, which facilitate entry into vulnerable cells [6]. Previous reports suggest that up to four distinct abrin and pulchellin protein isoforms exist. Both of these proteins have been shown to be toxic to human/primate cell lines and during mouse assays at varying levels, depending on the abrin [7] and pulchellin [8] isoforms isolated. Furthermore, agglutinin, an evolutionary variant of pulchellin and abrin, has been shown to be less toxic due to the lack of a critical hydrogen bond that causes a lower binding in between Abrin and 28S RNA [9]. From *Abrus precatorius*, the chemical isolation and initial cell toxicity testing of four distinct protein isoforms of abrin (abrin-a, -b, -c, and -d) and agglutinin was first published in the late 1970s and early 1980s [10,11]. The first successful cDNA conversion and cloning of these multiple sequences was carried out between 1991 and 1992 [12,13], providing the first genetic sequencing information on the coding of these toxin producing genes. In the mid-2000s, the Araújo Lab at the University of Sao Paulo cloned and expressed the A- and B-chains of pulchellin (orthologs of abrin genes) to test the toxicity of the compounds produced by the related species *Abrus pulchellus.* This group subsequently sequenced four mRNA prepropulchellin types [8,14,15]. Additional sequencing of standard plant genetic barcode regions for species identification and phylogenetic analysis are in abundance in GenBank as well, but complete genome sequencing and assembly of an *Abrus* genome has not been completed to date.

While the aforementioned research has provided critical information about the function and structure of these protein toxins, a genome from this plant genera has yet to be sequenced to determine the genetic basis for the generation of individual isoforms of these toxic compounds. To query the genomic structure and to understand the origin of different isoforms of these toxins, we sequenced leaf tissue of an *Abrus precatorius* cultivar grown at the University of Arizona. Whole genome sequencing data, using both long- and short-read technologies, were used to assemble the high-quality draft genome of *Abrus precatorius*. In addition, full-length transcript sequencing (Iso-Seq) [16] was performed to determine full-length, unique isoforms that encode the protein variants and aid in the full genome annotation of this plant. Due to the Select Agent status [1] and extreme toxicity of abrin, we chose to work only with leaf material, despite the understanding that the majority of the toxin production is found in the seeds.

From this work, we have identified multiple copies of abrin, abrin-like, and pulchellin-like genes within the assembled genome of *Abrus precatorius* cultivar 010017 and utilized full-length transcript sequencing (Iso-Seq; Pacific Biosciences RS-II platform) to identify genes that were expressed in leaves of this cultivar. The related toxin agglutinin was also queried for and identified.

## 2. Results

### Genome Sequencing, Assembly, and Annotation

The genome of *Abrus precatorius* cultivar 010017 was assembled into a 347 Mb genome containing 160 scaffolds (Table 1). Both genomic sequencing and full-length transcript sequencing were performed on the Pacific Biosciences RSII platform, which generates long-read data (average read length of 9374 bp). Genome annotation of the entire assembly was performed by the National Center for Biotechnology Information, utilizing the NCBI Eukaryotic Annotation Pipeline [17]. This pipeline utilized the full-length transcriptomic data collected to help inform accurate gene annotations and train gene-finding algorithms. The genome assembly and annotation is available in the NBCI Genbank Database (Accession number GCA_003935025.1 and Bioproject PRJNA482671).

**Figure 1:**
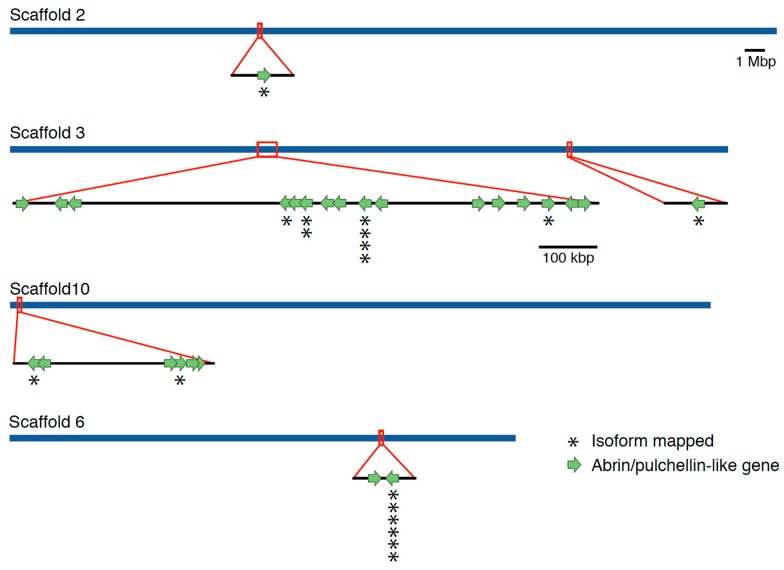
Location of abrin-like genes identified within the *Abrus precatorius* whole genome assembly. A total 26 Abrin-like genes were identified across four assembled scaffolds of the genome. Regions boxed in red are magnified to show the gene orientation in more detail. Nine of those genes that displayed active transcript evidence are highlighted with an “*”. For each “*” stacked an individual transcript was identified from the pool of full-length transcript sequencing data obtained in this study.

To identify all abrin-like, prepropulchellin-like (the gene that produces the pulchellin toxin), and agglutinin-like genes, BLASTn and BLASTp homology searches against the assembled *Abrus* genome were performed using published abrin and pulchellin sequences (Table S1). A total of 26 toxin-like genes or partial genes were identified in the *A. precatorius* 010017 genome across five distinct regions of the genome assembly (Figure 1). Of the 26 genes identified, 19 had intact open reading frames (Table S2).

A total of 18 abrin-like expressed isoforms were identified in the Iso-Seq transcriptomic assemblies. As the IsoSeq protocol allows sequencing of individual full-length gene expression transcripts, some transcripts are identical copies that were transcribed off of the same gene. Of the 18 isoforms identified, 9 of the 26 toxin-like genes identified within the genome assembly had one or more individual expressed isoforms that aligned to an abrin-like gene (Figure 1).

**Figure 2:**
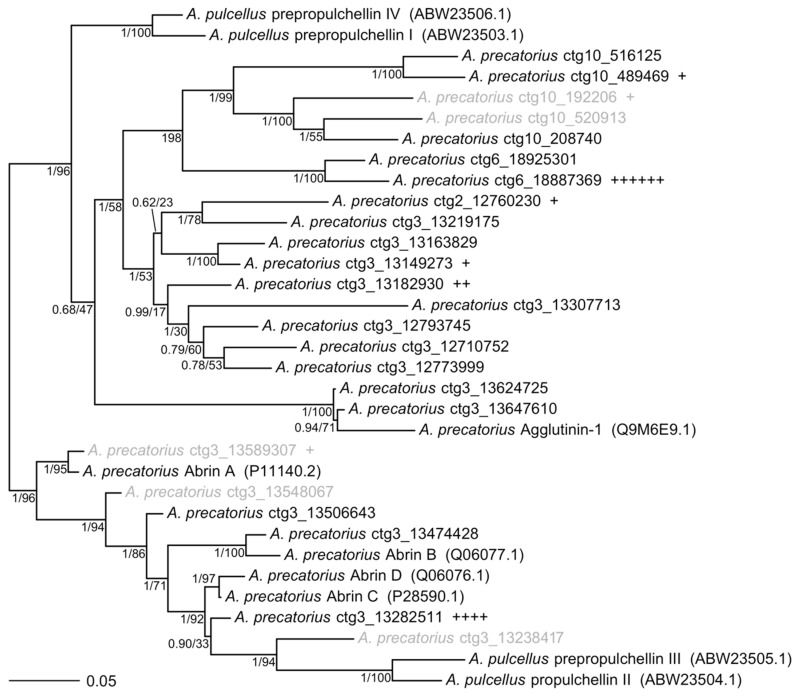
Unrooted phylogenetic tree of *Abrus* abrin and prepropulchellin protein sequences. Independent maximum likelihood (ML) and Bayesian analyses estimated the same tree topology with the exception of the poorly supported placement of ctg3_13282511. Numbers indicate ML bootstrap values and Bayesian posterior probabilities, respectively. Branch lengths and topology shown are from the Bayesian estimation. Novel sequences are denoted with contig number (“ctg”) and start codon position within genome assembly reported in this work (NCBI: GCA_003935025). Each instance of an individually sequenced isoform mapped to each sequence is denoted by a plus sign (18 total abrin-like isoforms were identified from the Iso-Seq sequencing). Accession numbers for reference sequences are included in parentheses. Sequences in gray indicate putative pseudogenes. Genes used in this comparison include Abrin A, Abrin B, Abrin C, Abrin D, prepropuchellin I–IV, and Agglutinin genes published on NCBI GenBank (Tables S1 and S2). The translated alignment file used to generate this tree is available (File S1).

To assess the relatedness of each toxin-like gene identified in the genome, all toxin-like gene sequences were aligned and compared to previously published mRNA transcripts of abrin, prepropulchellin, and agglutinin (Table S1). Amino acid translations of each gene (including genes that had frameshift mutations that were manually curated to create near full open reading frames (Table S2)) were utilized for this analysis. A phylogenetic tree was estimated from 24 complete, novel toxin-like and 9 reference protein sequences (Figure 2).

**Figure 3:**
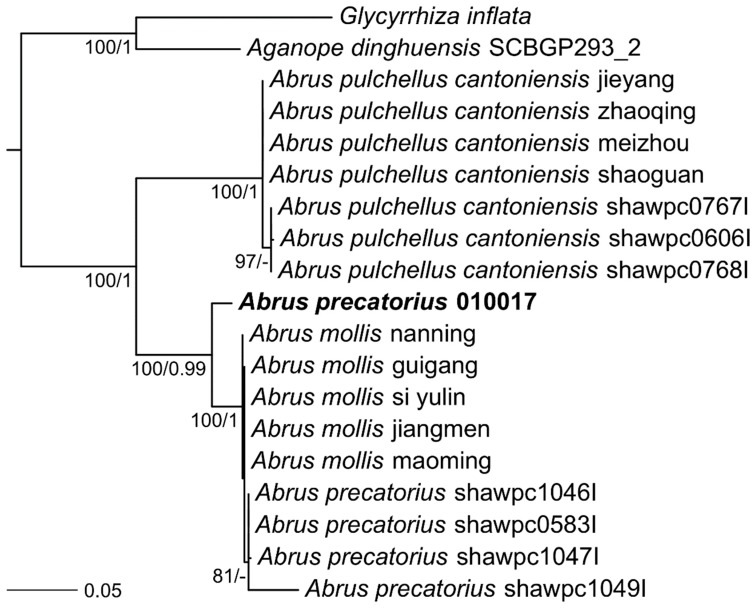
Phylogenetic tree of *Abrus* 18S rRNA sequences. Species sequenced here are emphasized. Numbers indicate independent ML bootstrap values and Bayesian posterior probabilities, respectively. Branch lengths shown are from the ML estimation. Outgroups, *Glycyrrhiza* (licorice) and *Aganope* (*Ostryocarpus*), used in this 18S analysis were two plants within the same family, Fabaceae, as *Abrus*.

To determine the relationship of this *A. precatorius* cultivar to other *Abrus* plants used in previous experimentation, the 18S rRNA sequence was used to build a phylogenetic tree of all available plants classified as a member of the *Abrus* genus. After sequencing and assembly of the *A. precatorius* 010017 genome, we identified the 18S ribosomal RNA encoding gene and aligned it to all other *Abrus* 18S sequences identified in GenBank (Figure 3). In this case, the Arizona 010017 cultivar of *A. precatorius* is strongly supported as sister to the *Abrus precatorius* and *Abrus mollis* clade. The *Abrus* cultivar used in this work did not form a monophyletic group with *Abrus pulchellus*.

## 3. Discussion and Conclusions

Here, we utilized full-length transcript sequencing (Iso-Seq) to isolate and sequence full-length RNA transcript isoforms from the leaves of *A. precatorius* 010017, in addition to sequencing and assembly of the genome of this plant. This is the first published attempt at examining the full genomic landscape of toxin genes in an *Abrus* cultivar. From this examination, a mixture of both abrin-like coding genes and prepropulchellin-like coding genes were identified as full-length transcripts using this methodology. Only a minority of toxin-like genes identified (9 of 26 genes) within the genome had evidence of expression in leaf tissue via full-length transcript sequencing and analysis. This is not necessarily surprising, due to the fact that only one time point was used for analysis and not all genes are expressed at all times and in all conditions or tissues—particularly, as the seeds of *Abrus* contain the majority of the abrin toxin. Additionally, if the transcript level of any of the other abrin or pulchellin gene(s) is low, it is likely that those associated transcripts were simply not captured in this Iso-Seq-based analysis. An alternative explanation suggests that many of the genes identified are actually non-functional or non-expressed pseudogenes.

The phylogeny of abrin-like genes indicates this *Abrus* isolate has orthologous sequences to Abrin A and Abrin B, but not Abrin C or Abrin D, though the Abrin A gene in this cultivar is likely a non-functional pseudogene due to a frameshift mutation. This suggests relatively rapid evolution of abrin genes throughout the *Abrus* clade, which has important implications for understanding abrin toxin evolution. Additional work, including sequencing of *Abrus mollis*, would be useful for identifying both abrin-like and prepropulchellin-like genes allowing for improved understanding of the toxin genes evolutionary history. Furthermore, this analysis has detected a large group of genes similar to prepropulchellin and Agglutinin-1, which are uncharacterized.

Until a better methodology to induce abrin expression in *A. precatorius* is attempted (i.e., utilizing seed material), we cannot expect to capture all abrin-like or prepropulchellin-like RNA transcripts that are truly expressed in this organism. Nevertheless, we were able to isolate full-length RNA transcripts, and novel abrin coding genes were identified within the high quality draft genome described in this paper.

Additional work with this new data may be of use for detection of transcription factor binding sites near the toxin-like genes, which may be useful in future investigations of gene activation properties. As we did not isolate any of the toxin-like gene products in question, we did not perform any testing of toxicity. It is likely fair to assume that toxicity levels to human and animal targets will vary within these gene products identified, based on the variations in toxicity to abrin and pulchellin shown previously [7,8]. Future approaches could include identifying *Abrus* cultivars that have closer 18S identity to those of plants that had the abrin or prepropulchellin genes cloned and toxicity characterized, to see if the same large cohort of toxin genes are again found in those strains.

## 4. Materials and Methods

### 4.1. Abrus Precatorius DNA Extraction and Preparation

Leaves of *Abrus precatorius* were flash-frozen in liquid nitrogen after propagation and harvesting by Matthew Johnson at the School of Plant Sciences at University of Arizona. Samples were shipped to Los Alamos National Lab on dry ice. The origin of this cultivar is from *Abrus precatorius* 010017 collected by S. Bentz in 2001 at Mount Carbine, S 16°31′, E 145°08′, Queensland, Australia. High Molecular weight genomic DNA was extracted from the leaf material using a modified CTAB (cetyl trimethylammonium bromide) extraction protocol. Genomic DNA was analyzed for DNA quality and subsequently sheared using the Megaruptor2 instrument (Diagenode, Ougrée, Belgium) with a target size of 60 kb. Multiple SMRT bell templates were prepared according to PacBio (Pacific Biosciences, Menlo Park, CA, USA) SMRT bell template prep protocol for templates longer than 15 kb. Libraries were size-selected on Blue Pippin (Sage Science, Beverly, MA, USA) instrument using marker S1, with 7 and 10 kb lower cutoff. Size selected DNA was collected and purified using AMPure PB beads (Beckman Coulter, Indianapolis, IN, USA). Sequencing primer was annealed and DNA polymerase bound to the SMRT bell templates. The libraries were sequenced on PacBio RSII DNA sequencer using C4/P6 chemistry.

### 4.2. Genome Sequencing

Of the 105 SMRT cells run on the RSII instrument, a total of 89 SMRT cells (consisting of a total of ~85 Gbp of data) were selected and used for assembly based on the highest quality metrics to assemble the *A. precatorius* draft genome. Raw read metrics of the SMRT cells quality consisted of a mean sub read length of 9374 bp, and an N50 was 14,043 bp. Illumina sequencing (2 × 300 paired end reads) was performed on the Illumina MiSeq (Illumina, San Diego, CA, USA) platform. A total of ~40 million sequencing reads with an average read length of 282 bp were used for subsequent steps after trimming with a q19 quality score cutoff using FaQC [18].

### 4.3. Genome Assembly and Polishing

A FALCON assembly (Version 0.7+, Pacific Biosciences, Menlo Park, CA, USA) [19] was completed using the sequencing data from all 89 cells. The seed coverage was set to 30x and the input genome size was set to 400,000 MB. The calculated length cutoff was 21,514 kb and the seed_N50 was 26,125 bp. The assembly contained 517 contigs with minimum length of 317 bp, maximum length of 14,274,906 bp, and N50 of 6,821,241 bp; 428 contigs were larger than 10 kb.

To improve the scaffolding of the *A. precatorius* genome, the Phase Genomics (Seattle, WA, USA) “Proximeta” service was used to improve the scaffolding of the *Abrus* genome assembly. This process uses Hi-C-based DNA crosslinking to more accurately determine scaffolding order of both isolated contigs and previously assembled scaffolds [20].

To further improve the quality of the scaffolded *Abrus precatorius* assembly, Illumina-based reads were utilized in two rounds of polishing in SMRT Link. Finally, the Illumina reads were mapped, using bowtie read mapper, back against all abrin coding regions to manually check the quality of the final assembly.

### 4.4. Full-Length Isoform Transcriptomic Sequencing and Analysis (Iso-Seq)

Flash-frozen leaves of *Abrus precatorius* were ground in liquid nitrogen and RNA was extracted using an Purelink Total RNA kit (Ambion, Austin, TX, USA). DNA was removed with on the column DNase treatment. Quality of extracted RNA was assessed on Qubit (Qubit RNA BR kit; Thermo Fischer, Waltham, MA, USA) and Bioanalyzer (RNA Pico kit; Agilent, Santa Clara, CA, USA). SMRT bell libraries were prepared according to the PacBio Isoform Sequencing (Iso-seq) protocol [21], utilizing the Clontech SMARTer PCR cDNA Synthesis Kit (Takara Bio, Kusatsu, Shiga Prefecture, Japan) and Blue Pippin Size-Selection System. The first step of the protocol was cDNA synthesis with adapters and PCR cycle optimization followed by large-scale PCR amplification. PCR products were then size partitioned on Blue Pippin, and large-scale PCRs performed to amplify various size products. SMRT bell libraries were prepared with size-selected PCR products, sequencing primer annealed and DNA polymerase bound to the libraries. The SMRT libraries were sequenced on the PacBio RSII using C4/P6 chemistry. A total of 20 SMRT cells were sequenced with a total output of 6.3 Gbases. Four SMRT cells were sequenced for the size range of 1–2 kb and 2–3 kb, respectively, and 16 cells were sequenced with the size-selected library of larger than 3 kb. The data were analyzed on Pacific Biosciences SMRT Portal informatics platform using the Iso-Seq application separately for each of the three pools, using default parameters. As a result of the analysis, low-quality and high-quality isoform fasta files were obtained. Blast analysis was performed to identify candidate isoforms with homology to published abrin/prepropulchellin gene products. All Iso-Seq reads were deposited in the NCBI SRA database under accession numbers SRX4674142 and SRX4674977–SRX4674981.

### 4.5. Phylogenetic Trees

We generated a phylogeny using Bayesian Markov chain Monte Carlo, implemented in MrBayes version 3.2.6 (University of California, Berkley, CA, USA) [22], and maximum-likelihood analyses, implemented in RAxML version 8.2.10 (Heidelberg Institute for Theoretical Studies, Germany) [23]. For the abrin-like toxin phylogeny, the data matrix included sequences for 33 terminal taxa, including 9 reference sequences. The sequence data consisted of 630 amino acids of abrin protein sequences. Five putative pseudogenes were included in the analysis. The determination of these pseudogenes was based off of the lack of fully intact open reading frames (Table S2), including frameshift and nonsense mutations. In order to include these five full-length sequences and display a historical view of abrin evolution, frameshifts and premature stop codons were removed. We repeated our analysis excluding these sequences; there were no strongly supported differences between the two analyses. Two partial sequences were excluded. For the 18S phylogeny, the data matrix included sequences for 19 terminal taxa, including 17 species, strains, and isolates of *Abrus*. The outgroup taxa represented two non-*Abrus* species, *Glycyrrhiza inflata* (GenBank: KY860932.1) and *Aganope dinghuensis* (GenBank: KP092718.1). The sequence data consisted of 724 nucleotides of 18S rRNA sequences. For both phylogenetic estimations, sequences were aligned using MAFFT version 7.394 [24] with the L-INS-i iterative refinement method. Four independent Bayesian runs of four chains each (three heated chains and one cold chain) were run for 5 × 10^7^ generations with a burn-in of 5 × 10^6^ generations. Trees were sampled every 100 generations. We considered the runs to have adequately sampled the solution space when the standard deviation of split frequencies was below 5 × 10^−3^. The trees where independently constructed using maximum likelihood (ML) methods with the rapid bootstrap analysis and the same partition scheme. Then, 350 ML replicate trees were used to estimate bootstrap support for the abrin-like phylogeny, while 700 ML replicate trees were used for the 18S phylogeny. For the abrin-like phylogeny, the revised Dayhoff rate model (JTTDCMut [25]) of protein evolution was selected by RAxML and implemented. For the 18S phylogeny, the general time-reversible model of nucleotide substation under the gamma-distributed model of rate heterogeneity with an estimate of proportion of invariable sites (GTR+Γ+I) was used for both Bayesian and ML analyses.

### 4.6. Description of the Cultivar

#### 4.6.1. Abrus Adanson. Familles des Plantes 2: 327, 511. 1763

A genus of ca. 17 species of annual or perennial climbing subshrubs, lianas, and herbs that are distributed from Africa and Madagascar to south and southeast Asia, and Australia. Introduced elsewhere.

#### 4.6.2. *Abrus precatorius* Linnaeus. Systema Naturae, ed. 12 2: 472. 1767

Common names include jequirity, ordeal bean, rosary pea, Indian licorice, crab’s eye, coral bead, and prayer bead.

#### 4.6.3. Basionym: *Glycine abrus* Linnaeus. Species Plantarum 2: 753-754. 1753.

Perennial climbing subshrubs or lianas to 5+ m with a deep root system. Stems herbaceous, dextrorsely twining. Pubescence of appressed, short, white hairs along stems, petioles, undersides of leaflets, inflorescence axes, and fruits. Leaves alternate, 2–7 cm, even-pinnate; stipules 3.5–4.5 mm, linear-lanceolate, deciduous; petiolate; stipels minute, slender, persistent. Leaflets 18–30(–40), 4–25 × 2.5–10 mm, oblong to oval or obovate, margins entire. Inflorescences axillary or terminal pseudoracemes, 4–12 cm, 20–50-flowered, crowded at distal end of axis; bracts 2, small, persistent; bracteoles 2, small, deciduous; pedicels 1.5–2 mm. Flowers papilionaceous; calyx 2–3 mm, nearly truncate or with shallow lobes to 0.2 mm; corolla 10–15 mm, white, pink-to-purplish or rarely yellow, banner ovate, short-clawed, wings long-clawed, keel curved, long-clawed; stamens 9, united into a sheath that is split distally, vexillary stamen absent, filaments filiform; ovary subsessile, style curved, stigma capitate. Fruits sessile or subsessile, oblong with a short beak, tan to brown, 2–4.5 × 1–1.5 cm, laterally compressed with two valves, dehiscent along one suture. Seeds nearly spherical to oval, lustrous red and black, (1–)3–7 per fruit, 5.5–7.5 × 4–5.5 mm, with a hard seed coat.

Native to Africa, Asia, and Australia, but widely introduced in tropical and subtropical regions around the world, where it is often aggressively invasive. In the US, *Abrus precatorius* is documented for Florida and Hawaii. Freezing winter temperatures preclude its survival across most of the US. Two subspecies are sometimes recognized. Subspecies *precatorius* is found across tropical Asia to Australia and subsp. *africanus* occurs in tropical Africa and Madagascar.

## Figures and Tables

**Table 1:** Genome assembly and annotation statistics for *Abrus precatorius.*

Genome Size	347,231,436 bp
GC%	31.8%
Number of contigs	344
Contig N50/L50	11,837,218/12
Number of scaffolds	160
Scaffold N50/L50	35,860,869/5
Predicted genes	29,216

## Supplementary Materials

The following are available online at https://www.mdpi.com/2072-6651/11/12/691/s1: Table S1. Accession number for reference sequences for abrin, pulchellin, and agglutinin analyses performed; Table S2. Detailed information for each toxin gene sequence identified in the *Abrus precatorius* genome; File S1. Aligned translated abrin sequences used for phylogenetic analysis presented in Figure 2.
