# Supplementary material for: Detection of Abrin-Like and Prepropulchellin-Like Toxin Genes and Transcripts Using Whole Genome Sequencing and Full-Length Transcript Sequencing of Abrus precatorius
Source: Toxins (Basel). 2019 Nov 25;11(12):691. doi: 10.3390/toxins11120691 (PMC6950105; doi:10.3390/toxins11120691)
Supplement: Supplementary file 1 [file toxins-11-00691-s001.zip › toxins-626222-Table S1 -conversion.pdf]

Supplementary Maaterials: Detection of Abrin-Like and Prepropulchellin-Like Toxin Genes and Transcripts Using Whole Genome Sequencing and Full-Length Transcript Sequencing of *Abrus precatorius*

Blake T. Hovde, Hajnalka E. Daligault, Erik R. Hanschen, Yuliya A. Kunde, Matthew B. Johnson, Shawn R. Starkenburg and Shannon L. Johnson

Table S1. Accession number for reference sequences for abrin, pulchellin, and agglutinin analyses performed.

Gene/Protein	Database	Accession Number
Abrin-a	UniProtKB/Swiss-Prot	P11140.2
Abrin-b	UniProtKB/Swiss-Prot	Q06077.1
Abrin-c	UniProtKB/Swiss-Prot	P28590.1
Abrin-d	UniProtKB/Swiss-Prot	Q06076.1
Agglutinin-1	UniProtKB/Swiss-Prot	Q9M6E9.1
prepropulchellin-I	NCBI Genbank	ABW23503.1
prepropulchellin-II	NCBI Genbank	ABW23504.1
prepropulchellin-III	NCBI Genbank	ABW23505.1
prepropulchellin-IV	NCBI Genbank	ABW23506.1
